# Lifetime HIV testing frequency among women in Sub-Saharan Africa: A DHS-based analysis using zero-inflated negative binomial regression

**DOI:** 10.1371/journal.pone.0354020

**Published:** 2026-07-16

**Authors:** Agazhe Aemro, Alebachew Ferede Zegeye, Enyew Getaneh Mekonen, Yilkal Abebaw Wassie, Mequannent Sharew Melaku, Basazinew Chekol Demilew

**Affiliations:** 1 Department of Medical Nursing, School of Nursing, College of Medicine and Health Sciences, University of Gondar, Gondar, Ethiopia; 2 Department of Surgical Nursing, School of Nursing, College of Medicine and Health Sciences, University of Gondar, Gondar, Ethiopia; 3 Department of Health Informatics, Institute of Public Health, College of Medicine & Health Sciences, University of Gondar, Gondar, Ethiopia; 4 Department of Anesthesia, College of Health Science, Debre Tabor University, Debre Tabor, Ethiopia; Bahir Dar University, ETHIOPIA

## Abstract

**Background:**

HIV testing is an essential component of HIV prevention and care, yet lifetime testing frequency remains low in sub-Saharan Africa (SSA). This study examines the factors influencing lifetime HIV testing frequency among reproductive-age women in SSA.

**Methods:**

We analyzed the most recent Demographic and Health Survey (DHS) data from nine sub-Saharan African countries, with an overall weighted sample of 158,722 women aged 15–49. The dependent variable was the lifetime number of HIV tests, and Zero-Inflated Negative Binomial (ZINB) regression was used. Model selection was based on Akaike Information Criterion (AIC) or Bayesian Information Criterion (BIC) and Vuong’s test, favoring the ZINB model over other count models. Adjusted incidence rate ratios (aIRRs) for the count and aOR for the inflation part with 95% confidence intervals (CIs) were reported, with statistical significance set at p < 0.05.

**Results:**

The mean number of lifetime HIV tests was 2.49 (SD = 7.67), with a median of 0 (IQR = 0–3). Key predictors of lifetime HIV testing included: age (30–39 years: aIRR = 2.94; 40–49 years: aIRR = 3.14), rural residence (aIRR = 1.10), current or former union (aIRR = 1.37 and 1.30, respectively), education (primary: aIRR = 1.19; secondary: aIRR = 1.35; higher: aIRR = 1.81), wealth (richer: aIRR = 1.30; richest: aIRR = 1.34), and employment (manual labor: aIRR = 1.21; skilled work: aIRR = 1.18). Other significant factors included: comprehensive HIV knowledge (aIRR = 1.37), STI awareness (aIRR = 2.21), media exposure (aIRR = 1.25), access to health facilities (aIRR = 1.26), number of sexual partners (1 partner: aIRR = 4.82; 2–3 partners: aIRR = 5.67; 4 or more partners: aIRR = 6.13), early sexual debut (aIRR = 0.61), and region (East Africa: aIRR = 2.82; Southern Africa: aIRR = 7.25). The inflation model showed very low odds of never testing among women with primary and secondary education, lower odds with media exposure (OR=0.40), but higher odds with higher education (OR=5.26) and rural residence (OR=1.93).

**Conclusions:**

This study indicated that over half of women of reproductive age in SSA have never tested for HIV. Addressing socio-economic, structural, and regional disparities, particularly in West Africa, is crucial, highlighting the need for targeted, equitable, and community-based strategies to expand testing coverage and promote repeat uptake.

## Introduction

HIV/AIDS remains one of the most significant public health challenges globally, particularly in Low- and Middle-income countries (LMICs) [1]. Although progress has been made in prevention and treatment, the burden is still substantial. According to the 2023 World Health Organization (WHO) and UNAIDS report, around 39 million people are living with HIV globally [2]. Sub-Saharan Africa remains the most affected region (about 67% of all people living with HIV). This reflects long-standing disparities in healthcare infrastructure, access to services, and social determinants of health [3].

Within SSA, women of reproductive age (15–49 years) carry a substantial share of the HIV burden, comprising approximately 63% of adults aged 15 and above living with the virus [4]. This heightened vulnerability arises from a combination of biological, social, and structural factors. Biologically, women have greater susceptibility to HIV infection due to anatomical and hormonal factors that increase transmission risk during heterosexual intercourse. Socially, practices such as early marriage, age-disparate relationships, and gender norms contribute to increased exposure and reduced activity in negotiating safe sex [4,5]. Structurally, limited access to healthcare services, pervasive stigma, gender-based violence, and sociocultural barriers impede timely HIV testing and treatment uptake [6]. These factors collectively hinder women’s ability to access prevention and care, perpetuating the epidemic within this demographic.

Lifetime HIV testing frequency is a key indicator for monitoring progress in HIV prevention and control efforts [6]. It refers to the frequency with which a woman has undergone HIV testing, reflecting the cumulative number of testing opportunities she has encountered throughout her life. Unlike single-time testing measures, lifetime testing frequency captures ongoing engagement with health systems, reflects repeated exposure to risk, and is essential for monitoring sustained prevention and treatment efforts [7]. Regular testing enables timely detection of new infections, supports linkage to care, and reduces onward transmission. Therefore, improving both the frequency and accessibility of HIV testing among women is vital for effective HIV control strategies in the region [8].

The WHO recommends universal HIV testing, particularly for high-risk populations including women. Testing at least once in a lifetime can lead to early diagnosis, timely treatment, and reduced transmission. However, despite expanded HIV testing efforts in sub-Saharan Africa, many women remain untested, reducing the effectiveness of HIV control strategies [9]. A multilevel analysis of data from 28 SSA countries showed that only 56.1% of women of reproductive age had ever been tested, with rates ranging from 86.9% in Zambia to just 6.1% in Chad [5]. Among antenatal care attendees, the average testing coverage was 62.87%, but as low as 5.73% in Mauritania [10]. In Mozambique, 57% of HIV-positive women had never been tested, revealing critical gaps in testing and diagnosis [11].Understanding lifetime number of HIV testing patterns is important in high-burden settings like SSA for identifying sociodemographic, economic, and geographic disparities, addressing service gaps, and informing targeted interventions to increase testing uptake and equity. Previous studies revealed significant variation in HIV testing uptake across SSA, influenced by individual, household, and community-level factors [5]. Common barriers to routine HIV testing include limited HIV knowledge, stigma, low autonomy, fear of partner violence, poor infrastructure, and test kit shortages. Moreover, age, education, residence, socioeconomic status, distance to health facilities, and lack of transportation are barriers for routine HIV testing. Addressing these challenges requires a comprehensive approach to improve HIV testing among high-risk groups, specifically reproductive-age women [12].

Although antenatal care (ANC) has played a significant role in increasing HIV testing among women [13], the overall frequency of lifetime testing in SSA remains unclear, particularly outside maternal health settings. Analyzing lifetime HIV testing patterns using nationally representative data like the Demographic and Health Surveys (DHS) is essential for identifying coverage gaps, promoting sustained HIV prevention, and addressing existing disparities. Therefore, this study aims to assess lifetime HIV testing frequency among women in SSA, determine key factors influencing testing frequency, and offer policy recommendations to enhance testing coverage. The findings will support evidence-based interventions to advance universal HIV testing for women in the region.

## Methods

### Study design and data source

This study employed a cross-sectional design using secondary data from the most recent DHS dataset conducted in sub-Saharan African countries. The DHS is a nationally representative household survey conducted approximately every five years, designed to collect data on a wide range of health indicators, including HIV testing behavior. These surveys follow a standardized methodology across countries, enabling cross-country comparability. We accessed the datasets from the DHS Program website (https://dhsprogram.com) and included the most recent DHS surveys (2021–2024) from selected SSA countries.

The variable “lifetime number of HIV tests” is a newly introduced indicator included in DHS Phase-8 (DHS-8); thus, only countries with DHS-8 data were eligible for inclusion. In SSA, thirteen countries have DHS-8 datasets available; however, four countries (The Gambia, Madagascar, Niger, and Rwanda) were excluded because the variable “lifetime number of HIV tests” in this countries contained no recorded data, possibly due to differences in questionnaire implementation or data availability. Ultimately, nine SSA countries with available and complete data on HIV testing frequency among women were included in the analysis **(see** Table 1**).**

**Table 1:** Description of study samples for the assessment of Lifetime number of HIV test among reproductive-age women in sub-Saharan Africa (n = 158,722).

S. No.	Country name	Region in Africa	Year of survey	Weighted sample	Percentage (%)
1.	Burkina Faso	West	2021	17,659	11.13
2.	Côte d’Ivoire	West	2021	14,877	9.37
3.	Democratic Republic of the Congo	Central	2023/24	27,583	17.38
4.	Ghana	West	2022/23	15,014	9.46
5.	Senegal	West	2023	16,583	10.45
6.	Kenya	East	2022	32,156	20.26
7.	Tanzania	East	2022	15,254	9.61
8.	Lesotho	Southern	2023/24	6,413	4.04
9.	Mozambique	Southern	2022/23	13,183	8.31
Total				158,722	100.00

### Study population

The study population consisted of reproductive-age women (15–49 years) who participated in the individual women’s survey (IR files) and had complete information on HIV testing history. According to DHS guidelines, women with missing data on whether they have ever had an HIV test are considered as never having been tested.

### Variables of the study

**Outcome variable:** The outcome variable for this study is the “lifetime number of HIV tests,” which refers to the total number of times a woman has ever been tested for HIV in her lifetime. It is derived from two variables: v781 (ever been tested for HIV: Yes or No) and v864 (number of HIV tests). Since v864 is only recorded for women who responded “Yes” to v781, those who answered “No” to v781 were assigned a value of zero tests. This approach resulted in a single variable with numeric values ranging from zero (for women who have never been tested) upward.

**Independent variables:** in this study, explanatory variables were selected based on findings from previous literatures, theoretical relevance, and availability in the DHS datasets. These includes:

**Socio-Demographic Factors**: Age group in years (15–19, 20–29, 30–39, 40–49), place of residence (urban, rural), marital status (never in union, currently in union/living together, formerly in union), educational status (no education, primary, secondary, higher), wealth index (poorest to richest), household headship (head, wife, child, other relatives/non-relative), occupation (had no work, agricultural, manual labor, white-collar/skilled), and region in Africa (West, East, Central, Southern). Wealth status was established through the pre-computed wealth index variable available in the DHS program dataset. The DHS wealth index is a constructed indicator of household living standards, developed through the principal components analysis (PCA) of data on asset ownership (e.g., television, car), housing characteristics, and access to water and sanitation facilities. A factor score was assigned to each asset based on the PCA, and the scores were standardized to have a mean of zero and a standard deviation of one. On the basis of these scores, the households were ranked and assigned into five equal population-based categories (Poorest, Poorer, Middle, Richer, and Richest).

**Reproductive, Behavioral, and Access-Related Factors**: Currently pregnant (yes, no), type of contraceptive use (none, traditional, modern method), age at first sex (never had sex, < 15 years, 15–17 years, 18–19 years, ≥ 20 years), lifetime number of sexual partners (zero, 1 partner, 2–3 partners, ≥ 4 partners), distance to health facilities (big problem, not a big problem), comprehensive HIV knowledge (yes, no), ever heard of STIs (yes, no), and media exposure (exposed: if exposed to at least one of reading newspapers, listening to the radio, or watching TV; otherwise not exposed). According to the DHS guideline, modern contraceptive methods include tubal ligation, voluntary surgical contraception, vasectomy, oral contraceptives pills, intrauterine device (IUD), Depo-Provera, implants, male and female condoms, lactational amenorrhea method (LAM), and emergency contraceptive pills. Traditional methods include rhythm/calendar method, withdrawal (coitus interruptus), and folkloric or traditional belief-based methods

### Data Processing and Analysis

The study utilized DHS data from selected Sub-Saharan African countries. Data were extracted, cleaned, and processed using STATA version 17. To account for the complex survey design and ensure representativeness at the national and regional levels, DHS-recommended procedures including sampling weights, stratification variables, and primary sampling unit (PSU) were incorporated throughout the analysis. Rather than fitting a multilevel model, a single-level zero-inflated negative binomial regression model was estimated, with cluster-robust standard errors at the PSU level (vce(cluster v001)) to account for within-cluster dependence. This adjusts the variance estimates for within-cluster dependence and provides valid statistical inference in the presence of intra-cluster correlation among respondents. Sampling weights were used to obtain representative population estimates. Descriptive statistics, including frequencies, percentages, means, and standard deviations, were computed to summarize the characteristics of the participants.

Given the count nature of the outcome variable (lifetime number of HIV tests), the presence of excess zeros (52.96% of participants reported zero tests), and with over-dispersion (variance: 58.82 vs. mean: 2.49), a Zero-Inflated Negative Binomial (ZINB) regression model was employed. The ZINB model consists of two parts: a negative binomial count model and a logit model for predicting the excess zeros. First, bivariate analysis was computed to identify the association between the outcome variable and possible independent variables. Then, independent variables with a p < 0.2 in the bivariate analysis were included in the multivariable ZINB model. Model comparison was performed using Akaike Information Criterion (AIC) or Bayesian Information Criterion (BIC), and Vuong’s test to justify the choice of the ZINB model over other count models.

All regression models were estimated using cluster-robust standard errors at the PSU level (vce(cluster v001)) in conjunction with sample weights to ensure robust statistical inference under the clustered survey design. Adjusted incidence rate ratios (aIRRs) and 95% confidence intervals (CIs) were reported, and level of statistical significance was set at p < 0.05.

To assess whether the higher HIV testing frequency in rural areas is a consistent statistical trend across all included countries or heavily driven by a specific country’s data, we performed a country-specific analysis. Separate ZINB regression models were computed for each included nation to determine the direction and consistency of the rural-urban differences across the included countries. Furthermore, the finding from a country-specific analysis was strengthened by conducting a country-by-place of residence interaction test.

### Ethical statement

The DHS datasets are publicly available upon request from the DHS Program (https://dhsprogram.com). Upon registration and approval of the data request, researchers can download datasets from the selected countries. Since the analysis is based on de-identified, publicly available secondary data, obtaining informed consent from participants or ethical approval from an institutional review board is not required.

## Results

### Socio-demographic Characteristics of Participants

In this study, a total weighted samples of 158,722 reproductive-age women were included in the analysis. The mean age of women was 28.78 years (SD = 9.66). About one-third of the participants were between 20 and 29 years of age. More than half lived in rural areas (56.22%), 60.06% were respond currently in union, and 24.64% had no education. In terms of relationship to the household head, about 15% of women were heads of their households. Regarding wealth of the participant’s, 16.72% were in the poorest quartile. Similarly, about 43% of the participants reported that they had no work, and 40.41% were from West-Africa **(see** Table 2**).**

**Table 2:** Socio-demographic characteristics of women in sub-Saharan Africa (n = 158,722).

S. No.	Variables	Categories	Frequencies	Percentages (%)
1.	Age of the respondent in years	Mean = 28.78; Standard deviation [SD] = 9.66		
2.	Age group of the respondents	15–19	33,652	21.20
		20–29	55,190	34.77
		30–39	42,277	26.64
		40–49	27,603	17.39
3.	Residence of the respondent	Urban	69,487	43.78
		Rural	89,235	56.22
4.	Respondent’s marital status	Never in union	48,565	30.60
		Currently in union	95,326	60.06
		Formerly in union	14,831	9.34
5.	Respondent’s educational status	No education	39,108	24.64
		Primary	44,253	27.88
		Secondary	62,103	39.13
		Higher	13,258	8.35
6.	Wealth index level	Poorest	26,537	16.72
		Poorer	28,275	17.81
		Middle	30,457	19.19
		Richer	34,420	21.69
		Richest	39,033	24.59
7.	Relationship to household head	Head	24,113	15.19
		Wife	68,089	42.90
		Child	46,474	29.28
		Other- or non-relatives	20,046	12.63
8.	Respondent’s occupation	Had no work	68,934	43.43
		Agricultural	27,768	17.49
		Manual labor	33,979	21.41
		White-collar/Skilled	28,041	17.67
9.	Region in Africa	West Africa	64,133	40.41
		East Africa	47,410	29.87
		Central Africa	27,583	17.38
		Southern Africa	19,596	12.35

### Reproductive, Behavioral, and access-related factors

This study reveals that the majority of women were not pregnant at the time of the survey (92.85%), and most participants reported not using any contraceptive method (70.07%), while a small proportion (4.10%) used traditional methods. Regarding age at first sexual intercourse, a significant portion initiated sex between 15 and 17 years (38.93%). Similarly, about one-third of women reported having only one lifetime sexual partner (33.73%), while 12.91% had four or more. When asked about access to health facilities, 38.94% perceived distance as a big problem, whereas the rest did not. Lastly, most participants lacked comprehensive knowledge of HIV (85.91%), although the majority had heard about Sexually-transmitted infections (STIs) (71.93%) and had media exposure (72.57%) **(see Table 3).**

**Table 3:** Reproductive, Behavioral, and access-related characteristics of reproductive-age women in sub-Saharan Africa (n = 158,722).

S. No.	Variables	Categories	Weighted Frequencies	Percentages (%)
1.	Currently pregnant	No	147,380	92.85
		Yes	11,342	7.15
2.	Type of contraceptive used	No method	111,223	70.07
		Traditional method	6,501	4.10
		Modern method	40,998	25.83
3.	Age at first sex	Never had sex	28,421	17.91
		<15 years	23,202	14.62
		15–17 years	61,787	38.93
		18–19 years	25,595	16.13
		≥20 years	19,717	12.42
4.	Lifetime number of sexual partners	Zero	39,205	24.70
		1 partner	53,535	33.73
		2–3 partners	45,489	28.66
		4 or more partners	20,493	12.91
5.	Distance to health facilities	Big problem	61,800	38.94
		Not a big problem	96,922	61.06
6.	Has comprehensive HIV knowledge	No	136,356	85.91
		Yes	22,366	14.09
7.	Ever heard of STIs	No	44,560	28.07
		Yes	114,162	71.93
8.	Media exposure status	Not exposed	43,538	27.43
		Exposed	115,184	72.57

### Lifetime HIV testing behavior of reproductive-age women in SSA

**Fig 1:**
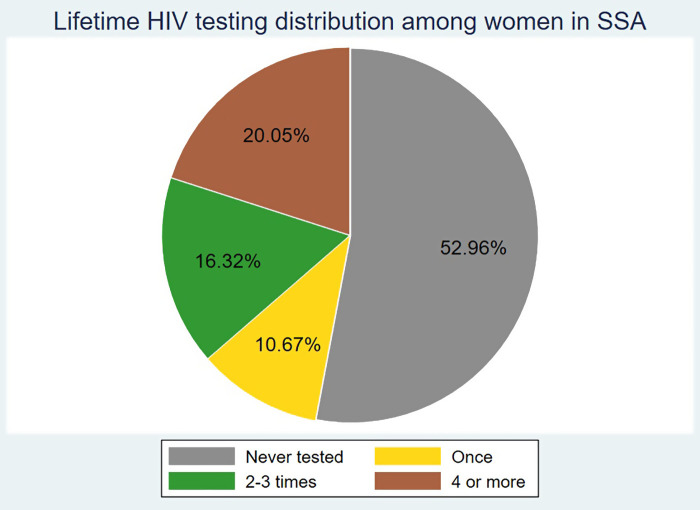
Lifetime HIV Testing Behavior of Reproductive-age Women in sub-Saharan Africa.

Among all women included in this study, 52.96% (95% CI: 52.72–53.21%) had never tested for HIV, 10.67% (95% CI: 10.52–10.82%) had been tested once, and 20.05% (95% CI: 19.85–20.25%) reported having undergone four or more lifetime HIV test **(see** Fig 1**).**

**Fig 2:**
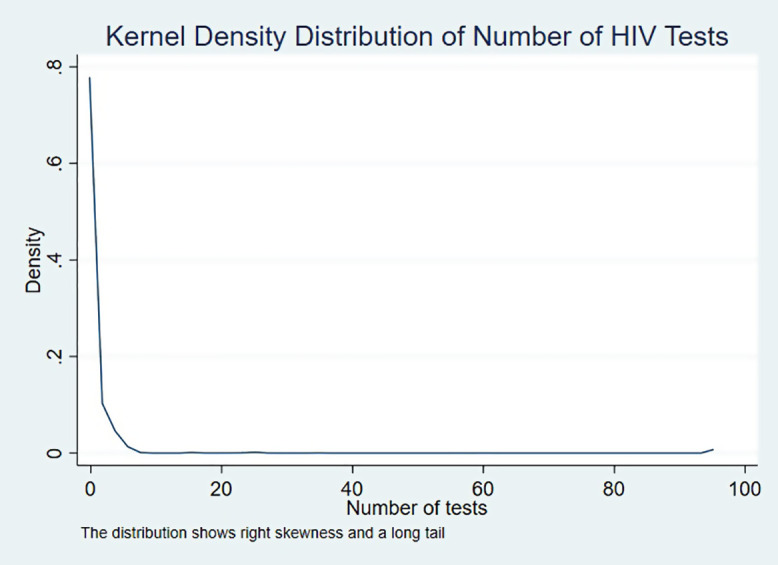
Kernel density plot of the number of lifetime HIV tests among reproductive-age women in SSA.

Additionally, the finding of this study revealed that the number of lifetime HIV tests ranged from 0 to 95, with a mean of 2.49 (SD = 7.67) and a median of 0 (Interquartile range [IQR] = 0–3). The variance was notably high at 58.82, indicating substantial variability in the number of HIV tests among women. Based on the median, at least half of the women had never been tested for HIV in their lifetime. To further illustrate the distribution of lifetime HIV testing, a kernel density plot of the outcome variable was generated. The distribution is highly right-skewed, with many women reporting zero tests and a long tail representing a small number of women who have many tests (see **Fig 2**).

### Bivariate Analysis of Factors Associated with Lifetime number of HIV Testing

Bivariate analysis was conducted to assess the unadjusted association between each independent variable and the lifetime number of HIV tests among reproductive-age women in sub-Saharan Africa. Significant differences in the number of lifetime HIV tests were observed across age groups, educational status, marital status, wealth status, relationship to household head, occupation, region, contraceptive use, age at first sex, lifetime number of sexual partners, distance to health facility, comprehensive HIV knowledge, and media exposure (*p* < 0.001). Additionally, pregnancy status at the time of the survey, having ever heard of STIs, and place of residence showed significant differences with p-values of 0.012, 0.014, and 0.164, respectively. These variables were subsequently considered for inclusion in the multivariable analysis.

### Summary of Model Comparison

To identify the most appropriate model for the count outcome variable (lifetime number of HIV tests), we compared the performance of Poisson, Negative Binomial (NB), Generalized Negative Binomial (GNB), and Zero-Inflated Negative Binomial (ZINB) models. The Poisson model exhibited overdispersion, as evidenced by a substantially large Pearson goodness-of-fit chi-square statistic (Pearson χ²/df > 1), indicating its unsuitability for the data. In contrast, the NB and GNB models showed improved fit, with identical log-likelihoods and lower AIC and BIC values, suggesting effective handling of overdispersion. Among all models, the ZINB model demonstrated the best fit, with the lowest AIC (526,626.6) and BIC (527115.6), and the highest log-likelihood (−263,264.4). The Vuong’s test comparing ZINB to NB was statistically significant (p < 0.001), supporting its superiority in accounting for excess zeros. Consequently, the ZINB model was selected as the final model for subsequent analyses **(see Table 4).**

**Table 4:** Model Comparison for Lifetime Number of HIV Tests among Reproductive-age Women in sub-Saharan Africa (n = 158,722).

Model	Log likelihood	AIC	BIC	Needs overdispersion test	Vuong’s test
Poisson	−485,683.06	971,440.1	971,809.2	**Yes** (χ^2^ = 2535273, df = 158685, p-value < 0.001)	N/A
Negative Binomial	−263,479.53	527,035.1	527,414.1	No	N/A
Generalized Negative Binomial	−263,479.53	527,035.1	527,414.1	No	N/A
Zero-inflated Negative Binomial	−263,264.4	526,626.6	527,115.6	No	Z = 10.38 p < 0.001

**Key: AIC** = *Akaike Information Criterion*; **BIC** = *Bayesian Information Criterion*; **df** = *Degrees of Freedom*; **χ²** = *Chi-squared statistic*; **N/A** = *Not Applicable*; **Z** = *Z-score* (or *Z-statistic)*

### Multivariable ZINB analysis of factors associated with lifetime number of HIV testing

The multivariable ZINB analysis identified several factors significantly associated with the lifetime number of HIV tests after adjusting for covariates. In the count model, compared to adolescents (15–19 years), women aged 30–39 (aIRR = 2.94; 95% CI: 2.64–3.28) and 40–49 (aIRR = 3.14; 95% CI: 2.83–3.49) had about three times the expected number of tests. Rural residents (aIRR = 1.10; 95% CI: 1.01–1.20) and women currently or formerly in union (aIRR = 1.37; 95% CI: 1.25–1.51 and 1.30; 95% CI: 1.17–1.44, respectively) had higher number of testing than urban residents and those never in union, respectively. Education and wealth showed positive associations with testing. Women with primary, secondary, and higher education had aIRRs of 1.19 (95% CI: 1.10–1.28), 1.35 (95% CI: 1.26–1.45), and 1.81 (95% CI: 1.46–2.24), respectively, compared to those with no education. Richer and richest wealth groups also had higher expected number of tests (aIRR = 1.30; 95% CI: 1.15–1.47 and 1.34; 95% CI: 1.19–1.50, respectively). Being the household head was associated with a 20% higher rate of lifetime HIV testing compared to other-/non-relatives (aIRR = 1.20; 95% CI: 1.06–1.35). Employment in manual labor (aIRR = 1.21; 95% CI: 1.14–1.29) and skilled work (aIRR = 1.18; 95% CI: 1.09–1.28) was linked with increase in the expected number of tests. Regionally, testing rates were higher in East (aIRR = 2.82; 95% CI: 2.64–3.01) and Southern Africa (aIRR = 7.25; 95% CI: 6.51–8.07), but 41% lower in Central Africa (aIRR = 0.59; 95% CI: 0.49–0.71) compared to West Africa.

Other significant predictors included current pregnancy (aIRR = 1.10; 95% CI: 1.03–1.16), modern contraceptive use (aIRR = 1.23; 95% CI: 1.17–1.29), comprehensive HIV knowledge (aIRR = 1.37; 95% CI: 1.24–1.51), STI awareness (aIRR = 2.21; 95% CI: 2.06–2.38), and media exposure (aIRR = 1.25; 95% CI: 1.16–1.34). Easier access to health facilities (aIRR = 1.26; 95% CI: 1.18–1.34) was also associated with more testing. Conversely, early sexual debut was linked to fewer lifetime tests. Women initiating sex before age 15 had a 39% lower expected number of tests (aIRR = 0.61; 95% CI: 0.48–0.77) than those who never had sex. Testing increased with the number of lifetime sexual partners, with aIRRs of 4.82 (95% CI: 3.69–6.30, one partner), 5.67 (95% CI: 4.32–7.46, 2–3 partners), and 6.13 (95% CI: 4.73–7.94, 4 or more partners).

In the inflation model identified factors that were significantly associated with the likelihood of never undergone HIV testing among women of reproductive age in SSA. Women residing in rural areas were nearly twice as likely to remain untested compared to urban residents (AOR = 1.93; 95% CI: 1.05–3.55). Women with primary and secondary education (OR ranging between 10 ⁻ ^8^ and 10^−7^) implied a near-zero likelihood of being untested. In contrast, higher education was linked to increased odds of being untested (AOR = 5.26; 95% CI: 2.41–11.48) compared to those with no education. Women in the poorer wealth quintile also had reduced odds being untested compared to the poorest category (OR = 0.40; 95% CI: 0.21–0.79). Additionally, perceiving distance to health facilities as not a big problem had substantially lower odds of being untested (OR = 2.56 × 10 ⁻ ^10^) and media exposure were significantly associated with lower odds of being untested (OR = 0.40; 95% CI: 0.23–0.70), suggesting that information exposure positively influences HIV testing behavior.

Moreover, the significant overdispersion (alpha = 1.44, 95% CI: 1.34–1.54, p < 0.001) supports the use of the negative binomial model over Poisson **(see** Table 5**).**

**Table 5:** Key determinants of Lifetime number of HIV testing among reproductive-age women in sub-Saharan Africa (n = 158,722).

Variables	Categories	Frequencies	aIRR	95% CI	P-value
Age group of the respondents	15–19	33,652	Ref		
	20–29	55,190	1.89	(1.71, 2.09)	0.000
	30–39	42,277	2.94	(2.64, 3.28)	0.000
	40–49	27,603	3.14	(2.83, 3.49)	0.000
Residence of the respondent	Urban	69,487	Ref		
	Rural	89,235	1.10	(1.01, 1.20)	0.032
Respondent’s marital status	Never in union	48,565	Ref		
	Currently in union	95,326	1.37	(1.25, 1.51)	0.000
	Formerly in union	14,831	1.30	(1.17, 1.44)	0.000
Respondent’s educational status	No education	39,108	Ref		
	Primary	44,253	1.19	(1.10, 1.28)	0.000
	Secondary	62,103	1.35	(1.26, 1.45)	0.000
	Higher	13,258	1.81	(1.46, 2.24)	0.000
Wealth index level	Poorest	26,537	Ref		
	Poorer	28,275	1.08	(1.00, 1.17)	0.047
	Middle	30,457	1.14	(1.04, 1.25)	0.006
	Richer	34,420	1.30	(1.15, 1.47)	0.000
	Richest	39,033	1.34	(1.19, 1.50)	0.000
Relationship to household head	Head	24,113	1.20	(1.06, 1.35)	0.003
	Wife	68,089	1.11	(0.99, 1.25)	0.075
	Child	46,474	1.06	(0.93, 1.21)	0.390
	Other-/non-relatives	20,046	Ref		
Respondent’s occupation	Had no work	68,934	Ref		
	Agricultural	27,768	1.03	(0.94, 1.13)	0.468
	Manual labor	33,979	1.21	(1.14, 1.29)	0.000
	White-collar/skilled	28,041	1.18	(1.09, 1.28)	0.000
Region in Africa	West Africa	64,133	Ref		
	East Africa	47,410	2.82	(2.64, 3.01)	0.000
	Central Africa	27,583	0.59	(0.49, 0.71)	0.000
	Southern Africa	19,596	7.25	(6.51, 8.07)	0.000
Currently pregnant	No	147,380	Ref		
	Yes	11,342	1.10	(1.03, 1.16)	0.004
Type of contraceptive used	No method	111,223	Ref		
	Traditional method	6,501	1.18	(0.93, 1.49)	0.168
	Modern method	40,998	1.23	(1.17, 1.29)	0.000
Age at first sex	Never had sex	28,421	Ref		
	<15 years	23,202	0.61	(0.48, 0.77)	0.000
	15-17 years	61,787	0.69	(0.57, 0.84)	0.000
	18-19 years	25,595	0.67	(0.55, 0.81)	0.000
	20 + years	19,717	0.62	(0.52, 0.75)	0.000
Lifetime number of sexual partners	Zero	39,205	Ref		
	1 partner	53,535	4.82	(3.69, 6.30)	0.000
	2–3 partners	45,489	5.67	(4.32, 7.46)	0.000
	4 or more	20,493	6.13	(4.73, 7.94)	0.000
Distance to health facilities	Big problem	61,800	Ref		
	Not a big problem	96,922	1.26	(1.18, 1.34)	0.000
Has comprehensive HIV knowledge	No	136,356	Ref		
	Yes	22,366	1.37	(1.24, 1.51)	0.000
Ever heard of STIs	No	44,560	Ref		
	Yes	114,162	2.21	(2.06, 2.38)	0.000
Media exposure status	Not exposed	43,538	Ref		
	Exposed	115,184	1.25	(1.16, 1.34)	0.000
**Inflation Model Results**					
**Variables**	**Categories**		**AOR**	**95% CIs**	**P-values**
Residence of the respondent	Urban		Ref		
	Rural		1.93	(1.05, 3.55)	0.033
Respondent’s educational status	No education		Ref		
	Primary		1.90 × 10 ⁻ ^8^	(1.40 × 10 ⁻ ^9^, 2.57 × 10 ⁻ ^7^)	0.000
	Secondary		1.07 × 10 ⁻ ^7^	(6.60 × 10 ⁻ ^9^, 1.73 × 10 ⁻ ^6^)	0.000
	Higher		5.26	(2.41, 11.48)	0.000
Wealth index level	Poorest		Ref		
	Poorer		0.40	(0.21, 0.79)	0.008
	Middle		0.63	(0.32, 1.21)	0.166
	Richer		0.78	(0.41, 1.49)	0.450
	Richest		0.91	(0.43, 1.92)	0.799
Distance to health facilities	Big problem		Ref		
	Not a big problem		2.56 × 10 ^− 10^	(9.75 × 10 ^− 11^, 6.73 × 10 ^− 10^)	0.000
Media exposure status	Not exposed		Ref		
	Exposed		0.40	(0.23, 0.70)	0.001
Dispersion parameter in the count model					0.000
	alpha		1.44	(1.34, 1.54)	

**Key**: ***aIRR*** *= Adjusted Incidence Rate Ratio;* ***CI*** *= Confidence Interval;* ***Ref*** *= Reference category*

### Country-specific analysis of rural-urban differences

The results of a country-specific ZINB regression analysis revealed substantial heterogeneity in the association between place of residence and lifetime HIV testing frequency across countries. Rural residence was associated with significantly higher HIV testing frequency in Kenya (aIRR = 1.16; 95% CI: 1.06–1.27), Lesotho (aIRR = 1.63; 95% CI: 1.30–2.05), and Senegal (aIRR = 1.34; 95% CI: 1.01–1.78). In contrast, fewer lifetime HIV tests were observed among rural residents in Burkina Faso (aIRR = 0.86; 95% CI: 0.75–0.99), while no statistical rural-urban difference was observed among the remaining countries. Moreover, the country-by-place of residence interaction term result in the pooled model was statistically significant (χ² = 139.62, p < 0.001), supporting significant cross-country heterogeneity between residence and lifetime HIV testing frequency **(S1 Table).**

## Discussion

This study assessed the lifetime number of HIV tests among reproductive-age women in sub-Saharan Africa using nationally representative DHS data. The mean number of lifetime HIV tests was 2.49 (SD = 7.67), while the median was 0 (IQR = 0–3), indicating a positively skewed distribution. This suggests that while most women had few or no tests, a smaller subset underwent repeated testing. Similar patterns have been observed in the region, where repeat testing remains limited despite expanded testing coverage [12,14]. Structural barriers such as stigma, restricted access to services, and low perceived risk of HIV may explain this disparity [15]. These findings highlight the importance of targeted strategies to enhance both initial uptake and continued participation in HIV testing, especially among women with limited access and awareness [16].

In the multivariable analysis, several factors were significantly associated with increased number of lifetime HIV testing. Compared to younger women, women aged 30–39 and 40–49 had about three times the lifetime testing rates (aIRR = 2.94 and 3.14, respectively). The results are consistent with findings from earlier studies in sub-Saharan Africa. This could be explained by the greater cumulative exposure of older women to healthcare services over time, including antenatal care, family planning programs, where HIV testing is routinely offered, as well as longer duration of sexual activity and changes in partner dynamics [5,14]. To account for the effects of cumulative exposure, we conducted an age-stratified zero-inflated negative binomial regression analysis. Within each age group, key predictors, including region, lifetime sexual partners, education, media exposure, and STI awareness, remained significantly associated with testing frequency. These results suggest that although longer exposure time contributes to higher testing rates among older women, behavioral, structural, and contextual factors independently influence HIV testing uptake across different age groups **(S2 Table).**

Another important factor affecting the lifetime HIV tests was place of residence. Women living in rural areas had a 10% higher rate of lifetime HIV testing compared to those in urbans (aIRR = 1.10). This result is somewhat surprising, as urban areas are typically assumed to have better access to HIV services. However, country-specific ZINB analysis alongside with residence-country interaction term demonstrated substantial rural-urban heterogeneity in HIV testing frequency across the included countries. Testing rate among rural residents was higher in Kenya, Lesotho, and Senegal, but fewer in Burkina Faso, and no significant difference was observed in other included countries. This suggests that the pooled estimate in SSA should be interpreted cautiously as the contextual and programmatic differences across the countries may influence HIV testing behavior **(S1 Table)**. Though our dataset did not include variables for mobile testing units, evidence from SSA shows that community-based and outreach strategies can effectively boost rural testing [17]. For example, in rural Kenya and Uganda, community health campaigns with home-based follow-up achieved up to 89% HIV testing coverage, demonstrating the success of bringing services directly to communities [18]. Systematic reviews also indicate that mobile, home-based, and other community testing approaches routinely reach populations outside of health facilities across multiple countries in the region [16]. Another possible explanation is that urban slum populations may remain underserved due to overcrowding, long waiting times, or stigma, which could limit repeated testing despite greater physical access. This finding warrants further investigation using longitudinal or programmatic data to clarify whether these patterns reflect true differences in service access or reporting behavior, considering country-level heterogeneity in HIV testing behavior.

Additionally, women who are currently or formerly in union reported higher rates of lifetime HIV testing compared to those who have never been in a union (aIRR = 1.37 and 1.30, respectively). This is consistent with earlier research indicating that marital status is linked to greater access to healthcare and increased use of services such as HIV testing. Women in unions often interact more with the healthcare system, particularly through services like antenatal care or family planning, where HIV testing is routinely offered [19]. Being in a relationship may also heighten perceived susceptibility to HIV due to sexual activity with a partner, thereby encouraging more frequent testing [20]. This highlights the importance of targeting HIV testing efforts toward both married and formerly married women to sustain consistent engagement with testing services.

This study reveals a positive association between educational attainment and the lifetime number of HIV testing uptake. Women with primary, secondary, and higher education had progressively higher lifetime HIV testing rates compared to those with no formal education (aIRR = 1.19, 1.35, and 1.81, respectively). This finding aligns with previous research indicating that education improves health literacy, which enhances understanding of HIV transmission and the importance of early detection [21,22]. Educated women are also more likely to utilize healthcare services, make informed health decisions, and access HIV testing [23,24]. Additionally, educational settings often serve as key platforms for disseminating HIV-related information, further influencing testing behaviors [25]. These findings highlight the role of education in shaping health behaviors and stress the need for interventions that improve educational access to enhance HIV testing rates and strengthen prevention efforts.

Wealth status showed a clear gradient, with women in the richer and richest quintiles reporting significantly higher lifetime HIV testing rates compared to those in the poorest quintile (aIRR = 1.30 and 1.34, respectively). This is consistent with literature indicating that higher socioeconomic status improves access to healthcare, health literacy, and the ability to seek care proactively [12]. Financial stability may enable women to overcome cost-related barriers, such as transportation or time off work, thereby facilitating more frequent HIV testing [26,27]. Similarly, employment in manual labor (aIRR = 1.21), and skilled work (aIRR = 1.18) was linked with increased testing uptake, aligning with studies from sub-Saharan Africa that show employed individuals are more likely to access HIV services than those unemployed [12,28]. Employment can enhance access through financial means and exposure to workplace-based health promotion, particularly in formal sectors. Evidence from Uganda further supports this, showing workplace HIV interventions improve testing uptake [29]. These findings highlight the importance of addressing economic barriers and leveraging employment structures to expand HIV testing coverage equitably.

HIV testing rates were significantly higher in East Africa (aIRR = 2.82) and Southern Africa (aIRR = 7.25) compared to West Africa, a disparity likely attributable to several interrelated factors. Countries in East Africa, such as Kenya and Tanzania, have benefited from extensive international support through programs and funding for HIV programs, contributing to stronger health systems, broader availability of testing services, and more proactive implementation of HIV-related interventions [30]. In Southern Africa, countries such as Lesotho and Mozambique have very high HIV prevalence globally (about 23% and 12% respectively), which has led to aggressive national testing policies supported by international partners such as the U.S. President’s Emergency Plan for AIDS Relief (PEPFAR) and the Global Fund [31–33]. These policies have integrated routine and provider-initiated testing into maternal reproductive health programs and emphasized community outreach, leading to widespread and repeated testing opportunities for women [34,35]. In contrast, many West African countries face persistent challenges such as weaker health infrastructure, lower prioritization of HIV testing, and sociocultural barriers that limit uptake of HIV tests [36,37].

Additionally, current pregnancy (aIRR = 1.10) and modern contraceptive use (aIRR = 1.23) were associated with increased likelihood of HIV testing, consistent with evidence that integrating testing into antenatal care and family planning services improves uptake among women [13,38]. These findings underscore the importance of region-specific strategies and the need to strengthen the integration of HIV testing within reproductive health services, particularly in settings where testing rates remain low.

The present study found that comprehensive HIV knowledge (aIRR = 1.37), STI awareness (aIRR = 2.21), and media exposure (aIRR = 1.25) were positively associated with lifetime HIV testing among reproductive-age women. These findings align with previous studies in sub-Saharan Africa, which consistently report that greater health knowledge and awareness enhance perceived risk and promote testing behavior. For example, a multi-country DHS analysis found that comprehensive HIV knowledge significantly predicted testing uptake, particularly among women with higher education and health literacy [39]. Similarly, STI awareness has been associated with increased health-seeking behavior, including HIV testing, due to improved risk perception and the recognition of early diagnosis benefits [12]. Media exposure further reinforces these effects by disseminating HIV-related information, reducing stigma, and encouraging service utilization, especially when messages are context-specific and culturally sensitive [40,41]. These results highlight the critical role of integrated health education and communication strategies in improving HIV testing uptake, particularly among underserved and high-risk groups.

This study found that easier access to health facilities (aIRR = 1.26) was associated with increased HIV testing, consistent with evidence that proximity and convenience significantly influence service uptake, especially in rural settings [8,42]. Testing also rose with the number of lifetime sexual partners, aIRRs of 4.82 (one partner), 5.67 (2–3 partners), and 6.13 (4 or more partners), reflecting prior findings that greater risk perception drives testing behavior [43]. Conversely, early sexual debut was linked to lower testing rates; women who initiated sex before age 15 had a 39% lower rate (aIRR = 0.61), aligning with studies showing that early initiation is associated with reduced autonomy, stigma, and limited access to health information and services [44]. These findings underscore the need to enhance service accessibility and implement youth-focused strategies to reach under-tested, high-risk groups.

The inflation component of the ZINB model identified factors associated with the likelihood of being in the “never tested” group among reproductive age women in SSA. Women with primary and secondary education had extremely low odds of being in the never tested group (OR as low as 10 ⁻ ⁸) compared to those with no education. This indicates that even with minimal schooling greatly enhances awareness of HIV testing and access to HIV testing. This is also supported by the result from the count section that HIV testing increased with education. In contrast, women with higher education showed increased odds of being in the never testing groups (OR = 5.26). This may not indicate lack of awareness, but rather differences in risk perception, preference for private or self-testing options not captured in survey data, or lower exposure to community-based campaigns that often target less-educated groups. Overall, these findings suggest that higher education does not always translate into more frequent HIV testing and that tailored strategies are needed to engage women across all educational levels.

Similarly, the inflation section revealed that media exposure was associated with a 60% reduction in in the odds of never testing (OR = 0.40), highlighting the role of media in disseminating HIV-related information and motivation for testing. In addition, women who reported that distance to health facilities was not a big problem had substantially lower odds of belonging to the never-testing group (OR = 2.56 × 10^−10^). This highlights the crucial importance of geographical accessibility in promoting HIV testing uptake.

This study has its own limitations. First, the cross-sectional nature of the DHS data limits the ability to establish temporal relationships between predictors and HIV testing frequency. Second, the lifetime number of HIV tests relies on self-reported information and spans a long recall period, which may introduce potential recall bias, particularly among older women. Underreporting, heaping, or rounding of the number of tests could further distort associations in the regression estimates. Self-reported data are also prone to social desirability biases, particularly for sensitive behaviors such as sexual history or HIV testing. Third, the data do not provide information on the results of previous tests or whether women stopped testing after receiving a positive result, and HIV status at the time of the survey may not accurately reflect earlier status; the analysis therefore examines testing frequency independent of test outcomes. Fourth, the analysis was limited to women aged 15–49, excluding early adolescents and older women who may also engage in risk behaviors but are not represented in the DHS sampling frame. ICCs and between-cluster variance could not be calculated since a multilevel model was not fitted. Finally, unmeasured contextual factors, such as the local density of healthcare facilities or the intensity of community-level HIV awareness campaigns, along with the exclusion of countries lacking complete outcome data, may have introduced residual confounding and selection bias and reduced the overall comprehensiveness of regional comparisons.

## Conclusion

This study reveals that over half of women of reproductive age in sub-Saharan Africa have never undergone HIV test, with most having tested only once or not at all. Lifetime testing is influenced by demographic, socioeconomic, behavioral, and geographic factors. Women who are older, more educated, and richer, as well as those who have better access to health information and services, have higher testing rates, while adolescents and women who are structurally and informationally disadvantaged are left behind. Large geographic disparities continue to exist, especially between West Africa and Southern and Eastern Africa. To reduce these gaps, HIV prevention strategies should prioritize expanding testing access for under-tested groups through integrated reproductive health and community-based approaches. Improving education, increasing the role of media in HIV awareness, and improving physical access to HIV testing facilities is important to encourage repeat testing and lower the percentage of women who remain untested. Scaling up the reach of context-specific interventions in low-testing regions, especially West Africa, will be essential to improve overall testing uptake and contribute to broader HIV prevention and care efforts.

## Supporting information

S1 File Anonymized dataset for the Lifetime HIV testing frequency in SSA. (CSV)

S1 Table Country-specific Zero-inflated negative binomial regression result showing the association between rural residence and HIV testing frequency among women in SSA. (DOCX)

S2 Table Age-stratified zero-inflated negative binomial regression analysis of factors associated with lifetime HIV testing frequency among women in SSA. (DOCX)

## Abbreviations

AIC: Akaike Information Criterion
AIDS: Acquired Immune Deficiency Syndrome
aIRR: Adjusted Incidence Rate Ratio
ANC: Antenatal Care
BIC: Bayesian Information Criterion
CIs: Confidence Intervals
DHS: Demographic and Health Surveys
HIV: Human Immunodeficiency Virus
IQR: Interquartile Range
LMICs: Low- and Middle-Income Countries
SD: Standard Deviation
SSA: Sub-Saharan Africa
STIs: Sexually Transmitted Infections
UNAIDS: Joint United Nations Programme on HIV/AIDS
WHO: World Health Organization
ZINB: Zero-Inflated Negative Binomial
