# Supplementary material for: Lifetime HIV testing frequency among women in Sub-Saharan Africa: A DHS-based analysis using zero-inflated negative binomial regression
Source: PLoS One. 2026 Jul 16;21(7):e0354020. doi: 10.1371/journal.pone.0354020 (PMC13374885; doi:10.1371/journal.pone.0354020)
Supplement: S1 Table — (DOCX) [file pone.0354020.s002.docx]

**Supplementary Table 1**: Country-specific Zero-inflated negative binomial regression result showing the association between rural residence and HIV testing frequency among women in SSA.

| Country | Weighted sample | aIRR (Rural vs Urban) | 95% CI | P-value | Interpretation |
| --- | --- | --- | --- | --- | --- |
| Burkina Faso | 17,659 | 0.86 | 0.75, 0.99 | **0.041** | Rural lower testing |
| Democratic Republic of Congo | 27,583 | 1.01 | 0.75, 1.36 | 0.956 | No difference |
| Côte d'Ivoire | 14,877 | 0.99 | 0.82, 1.21 | 0.990 | No difference |
| Ghana | 15,014 | 0.96 | 0.85, 1.07 | 0.446 | No difference |
| Kenya | 32,156 | 1.16 | 1.06, 1.27 | **0.001** | Rural higher testing |
| Lesotho | 6,413 | 1.63 | 1.30, 2.05 | **0.000** | Rural higher testing |
| Mozambique | 13,183 | 0.98 | 0.88,1.10 | 0.747 | No difference |
| Senegal | 16,583 | 1.34 | 1.01, 1.78 | **0.044** | Rural higher testing |
| Tanzania | 15,254 | 0.99 | 0.85, 1.15 | 0.880 | No difference |

**Footnote**: Country-by-residence interaction: χ² = 139.62, p < 0.001.
