# Supplementary material for: Lifetime HIV testing frequency among women in Sub-Saharan Africa: A DHS-based analysis using zero-inflated negative binomial regression
Source: PLoS One. 2026 Jul 16;21(7):e0354020. doi: 10.1371/journal.pone.0354020 (PMC13374885; doi:10.1371/journal.pone.0354020)
Supplement: S2 Table — (DOCX) [file pone.0354020.s003.docx]

**Supplementary Table 2**. Age-stratified zero-inflated negative binomial regression analysis of factors associated with lifetime HIV testing frequency among women in SSA

| **Variables** | **Categories** | **15–19 years** | **20–29 years** | **30–39 years** | **40–49 years** |
| --- | --- | --- | --- | --- | --- |
|  |  | aIRR (95% CI) | aIRR (95% CI) | aIRR (95% CI) | aIRR (95% CI) |
| Residence of the respondent | Urban | Ref | Ref | Ref | Ref |
|  | Rural | 1.24 (1.02, 1.49)* | 1.18 (1.06, 1.30)* | 0.99 (0.89, 1.09) | 0.87 (0.74, 1.02) |
| Respondent’s marital status | Never in union | Ref | Ref | Ref | Ref |
|  | Currently in union | 2.23 (1.78, 2.79)** | 1.44 (1.30, 1.59)** | 0.93 (0.81, 1.07) | 0.68 (0.49, 0.95)* |
|  | Formerly in union | 1.81 (1.18, 2.77)* | 1.34 (1.16, 1.54)** | 1.00 (0.87, 1.16) | 0.68 (0.50, 0.91)* |
| Respondent’s educational status | No education | Ref | Ref | Ref | Ref |
|  | Primary | 1.09 (0.85, 1.39) | 1.19 (1.09, 1.31)** | 1.17 (1.06, 1.28)* | 1.31 (1.13, 1.53)** |
|  | Secondary | 1.84 (1.43, 2.36)** | 1.44 (1.31, 1.57)** | 1.21 (1.09, 1.35)** | 1.47 (1.28, 1.68)** |
|  | Higher | 1.53 (0.98, 2.39) | 2.00 (1.72, 2.33)** | 1.84 (1.37, 2.46)** | 1.38 (1.16, 1.65)** |
| Wealth index level | Poorest | Ref | Ref | Ref | Ref |
|  | Poorer | 0.98 (0.80, 1.21) | 1.09 (0.99, 1.21 | 0.92 (0.82, 1.02) | 1.01 (0.86, 1.19) |
|  | Middle | 1.00 (0.78, 1.29) | 1.22 (1.09, 1.37)* | 0.92 (0.82, 1.03) | 0.99 (0.87, 1.13) |
|  | Richer | 0.99 (0.75, 1.32) | 1.37 (1.21, 1.54)** | 1.02 (0.88, 1.17) | 1.19 (0.95, 1.48) |
|  | Richest | 0.92 (0.68, 1.26) | 1.38 (1.19, 1.60)** | 1.10 (0.97, 1.24) | 1.13 ()0.93, 1.37 |
| Relationship to household head | Head | 1.41 (1.03, 1.94)* | 1.14 (1.01, 1.29)* | 0.91 (0.79, 1.04) | 1.20 (0.98, 1.47) |
|  | Wife | 0.79 (0.57, 1.08) | 1.07 (0.96, 1.19) | 0.95 (0.82, 1.10) | 1.13 (0.92, 1.40) |
|  | Child | 0.93 (0.74, 1.15) | 1.04 (0.93, 1.16) | 0.85 (0.72, 0.99)* | 0.98 (0.78, 1.23) |
|  | Other-/non-relatives | Ref | Ref | Ref | Ref |
| Respondent’s occupation | Had no work | Ref | Ref | Ref | Ref |
|  | Agricultural | 0.82 (0.71, 0.94)* | 1.01 (0.93, 1.09) | 0.90 (0.79, 1.03) | 1.23 (1.07, 1.43)* |
|  | Manual labor | 1.20 (1.05, 1.38)* | 1.29 (1.19, 1.42)** | 1.09 (0.97, 1.23) | 1.22 (1.09, 1.37)** |
|  | White-collar/skilled | 1.17 (0.97, 1.41) | 1.35 (1.23, 1.47)** | 1.00 (0.85, 1.18) | 1.17 (1.02, 1.33)* |
| Region in Africa | West Africa | Ref | Ref | Ref | Ref |
|  | East Africa | 5.55 (4.84, 6.35)** | 3.06 (2.83, 3.31)** | 2.17 (1.97, 2.39)** | 2.36 (2.10, 2.65)** |
|  | Central Africa | 0.37 (0.29, 0.47)** | 0.53 (0.45, 0.62)** | 0.61 (0.46, 0.81)* | 0.60 (0.48, 0.75)** |
|  | Southern Africa | 16.91 (13.6, 21.01)** | 6.50 (5.81, 7.27)** | 5.03 (4.49, 5.64)** | 5.96 (5.11, 6.96)** |
| Currently pregnant | No | Ref | Ref | Ref | Ref |
|  | Yes | 1.03 (0.86, 1.23) | 1.13 (1.02, 1.25)* | 1.09 (0.99, 1.20) | 1.07 (0.89, 1.29) |
| Type of contraceptive used | No method | Ref | Ref | Ref | Ref |
|  | Traditional method | 1.29 (0.97, 1.72) | 0.95 (0.81, 1.12) | 1.41 (0.97, 2.03) | 1.06 (0.94, 1.21) |
|  | Modern method | 1.45 (1.24, 1.71)** | 1.23 (1.14, 1.33)** | 1.29 (1.19, 1.39)** | 1.19 (1.08, 1.31)** |
| Age at first sex | Never had sex | Ref | Ref | Ref | Ref |
|  | <15 years | 0.31 (0.21, 0.44)** | 0.54 (0.42, 0.70)** | 0.53 (0.42, 0.67)** | 0.46 (0.36, 0.59)** |
|  | 15-17 years | 0.35 (0.24, 0.50)** | 0.59 (0.46, 0.76)** | 0.59 (0.47, 0.74)** | 0.62 (0.49, 0.77)** |
|  | 18-19 years | 0.30 (0.21, 0.44)** | 0.53 (0.41, 0.68)** | 0.61 (0.48, 0.76)** | 0.67 (0.52, 0.86)* |
|  | 20+ years | ----- | 0.51 (0.39, 0.65)** | 0.52 (0.41, 0.66)** | 0.60 (0.47, 0.76)** |
| Lifetime number of sexual partners | Zero | Ref | Ref | Ref | Ref |
|  | 1 partner | 8.11 (5.43, 12.12)** | 6.48 (4.77, 8.80)** | 3.78 (2.34, 6.13)** | 2.72 (1.49, 4.99)* |
|  | 2–3 partners | 11.94 (8.12, 17.57)** | 7.56 (5.53, 10.32)** | 4.42 (2.77, 7.04)** | 3.01 (1.67, 5.42)** |
|  | 4 or more | 14.05 (8.99, 21.94)** | 9.10 (6.60, 12.54)** | 4.47 (2.87, 6.96)** | 2.92 (1.61, 5.29)** |
| Distance to health facilities | Big problem | Ref | Ref | Ref | Ref |
|  | Not a big problem | 1.37 (1.21, 1.56)** | 1.27 (1.18, 1.36)** | 1.22 (1.12, 1.32)** | 0.99 (0.86, 1.14) |
| Comprehensive HIV knowledge | No | Ref | Ref | Ref | Ref |
|  | Yes | 1.94 (1.65, 2.29)** | 1.18 (1.08, 1.28)** | 1.28 (1.15, 1.42)** | ------- |
| Ever heard of STIs | No | Ref | Ref | Ref | Ref |
|  | Yes | 2.22 (1.88, 2.62)** | 2.08 (1.86, 2.32)** | 2.21 (2.00, 2.44)** | 2.29 (2.02, 2.61)** |
| Media exposure status | Not exposed | Ref | Ref | Ref | Ref |
|  | Exposed | 1.47 (1.26, 1.72)** | 1.23 (1.13, 1.33)** | 1.15 (1.05, 1.27)* | 1.16 (1.01, 1.33)* |

**Key**: “-------” means not estimable.

* Significant at a p-value of < 0.05; ** Significant at a p-value of < 0.001
